# Maximal stomatal conductance to water and plasticity in stomatal traits differ between native and invasive introduced lineages of *Phragmites australis* in North America

**DOI:** 10.1093/aobpla/plw006

**Published:** 2016-01-27

**Authors:** V. Douhovnikoff, S. H. Taylor, E. L. G. Hazelton, C. M. Smith, J. O'Brien

**Affiliations:** 1 Bowdoin College, 6500 College Station, Brunswick, ME 04011, USA; 2 Department of Watershed Sciences, Ecology Center, Utah State University, Logan, UT 84322, USA

**Keywords:** Clonal plant, invasive, *Phragmites*, plasticity, stomata

## Abstract

The fitness costs of reproduction by clonal growth can include a limited ability to adapt to environmental and temporal heterogeneity. Paradoxically, some facultatively clonal species are not only able to survive, but colonize, thrive and expand in heterogeneous environments. This is likely due to the capacity for acclimation (*sensu stricto*) that compensates for the fitness costs and complements the ecological advantages of clonality. Introduced *Phragmites australis* demonstrates great phenotypic plasticity in response to temperature, nutrient availability, geographic gradient, water depths, habitat fertility, atmospheric CO_2_, interspecific competition and intraspecific competition for light. However, no *in situ* comparative subspecies studies have explored the difference in plasticity between the non-invasive native lineage and the highly invasive introduced lineage. Clonality of the native and introduced lineages makes it possible to control for genetic variation, making *P. australis* a unique system for the comparative study of plasticity. Using previously identified clonal genotypes, we investigated differences in their phenotypic plasticity through measurements of the lengths and densities of stomata on both the abaxial (lower) and adaxial (upper) surfaces of leaves, and synthesized these measurements to estimate impacts on maximum stomatal conductance to water (*g*_wmax_). Results demonstrated that at three marsh sites, invasive lineages have consistently greater *g*_wmax_ than their native congeners, as a result of greater stomatal densities and smaller stomata. Our analysis also suggests that phenotypic plasticity, determined as within-genotype variation in *g*_wmax_, of the invasive lineage is similar to, or exceeds, that shown by the native lineage.

## Abstract (precis)

Using previously identified Phragmites clonal genotypes we investigated differences in their phenotypic plasticity through measurements of the lengths and densities of stomata on both the abaxial (lower) and adaxial (upper) surfaces of leaves, and synthesized these measurements to estimate impacts on maximum stomatal conductance to water (*g*_*wmax*_). Results demonstrated that at three marsh sites invasive lineages have consistently greater *g*_*wmax*_ than their native congeners, as a result of greater stomatal densities and smaller stomata. Our analysis also suggests that phenotypic plasticity, determined as within genotype variation in *g*_*wmax*_*,* of the invasive lineage is similar to, or exceeds that shown by the native lineage.

## Introduction

The capacity for clonal growth is often given as an explanation for the invasive character of many introduced species (Thompson *et al*. 1995). Clonal growth affords species a capacity for reproduction despite small initial population sizes. It also offers competitive advantages such as the ability to nurse new ramets (sprouts), share resources between ramets and avoid the costly risks involved in sexual reproduction. However, the fitness costs of reproduction by clonal growth can include a limited ability to adapt to environmental and temporal heterogeneity (Alpert and Simms 2002). Recombination of genetic material and associated natural selection are not available for the rapid innovation and trial of new genotypes in clones, suggesting that the range of habitats invaded by clonal lineages should be more limited than that inhabited by competitors exhibiting more frequent sexual reproduction. Paradoxically, some facultatively clonal species are not only able to survive, but colonize, thrive and expand in heterogeneous environments. What factors underlie the success of particularly invasive clonal lineages? We hypothesize that these lineages are able to compete with, and ultimately outcompete, species with more diverse gene pools, greater rates of recombination or longer history of local adaptation, through the process of acclimation (*sensu stricto*) and a potentially greater range of phenotypic plasticity, which compensates for the fitness costs and complements the ecological advantages of clonality.

*Phragmites australis* is a large stature clonal grass that is found in a wide range of wetland and marsh-like ecosystems and occurs on every continent but Antarctica. In North America, several lineages have been recognized, while two are most prevalent: *P. australis* (Trin. × Steud.) is an invasive lineage (introduced), and *P. australis* subspecies *americanus* (Saltonstall, PM Peterson and Soreng) is a native lineage (native) (Saltonstall *et al*. 2004). Both the native and introduced lineages have the capacity for extensive clonal growth (Douhovnikoff and Hazelton 2014). However, the introduced lineage is expanding its range and outcompeting many native species across a broad range of local conditions and wetland types throughout North America.

Introduced *P. australis* demonstrates great phenotypic plasticity in response to temperature and nutrient availability (Eller and Brix 2012), geographic gradient (Bastlova *et al*. 2004), water depths (Vretare *et al*. 2001), habitat fertility (Clevering 1999), atmospheric CO_2_ (Mozdzer and Megonigal 2012), interspecific competition (Bellavance and Brisson 2010) and intraspecific competition for light (Bellavance and Brisson 2010). However, the majority of prior work focussed on common garden studies with the European ancestral lineage, and not plants collected in North America. Further, no *in situ* comparative lineage studies have explored the difference in plasticity between the invasive introduced and non-invasive native lineages (reviewed in Mozdzer *et al*. 2013). Despite the obvious comparative potential, such closely related groups have rarely been examined with respect to the ecology of invasion. Among 93 comparative studies of plasticity in invasive plants identified by Palacio-López and Gianoli (2011), the closest shared taxon was at the genus level.

In addition to closely related (conspecific) lineages, the clonality of the native and introduced lineages make *P. australis* a unique system for the comparative study of phenotypic plasticity. Phenotype is a result of genetic (G) × environmental (E) interactions (Via and Lande 1985). Clonal plants are powerful model systems as they control for genetics (G). Assuming moderate mutation rates and developmental differences among compared groups, observed variation would largely be explained by plastic responses to environmental (E) conditions. Naturally occurring replicates (ramets) of a given genotype (genet) make it possible to measure and compare the reaction norms within and between genotypes permitting a better understanding of the role plasticity plays in plant ecology (Douhovnikoff and Dodd 2015) from the ramet to the lineage scale (Gianoli and Valladares 2012).

The size and spacing of stomata on leaves are simple measurements that provide a strong framework within which to explore phenotypic plasticity linked with physiological performance (Hetherington and Woodward 2003). Stomata permit and regulate gas exchange between the inner plant and the atmosphere, facilitating the exchange of gases necessary for photosynthesis and transpiration. In moving air, stomatal conductance is the principal control over leaf gas exchange with direct consequences for both leaf metabolism and energy balance (Schulze *et al*. 1994). Stomatal morphometrics provide an accurate representation of the capacity for leaf gas exchange through the calculation of maximal conductance (*g*_max_, Dow *et al*. 2014), which incorporates the influences of stomatal pore area and pore depth (Brown and Escombe 1900). The multi-dimensional framework for the assessment of stomatal variation provided by *g*_max_ has been used to demonstrate both heritable variation and environmental plasticity (Franks *et al*. 2009; Fanourakis *et al*. 2015). Differences in stomatal morphometrics have previously been identified for *P. australis* lineages (Hansen *et al*. 2007; Saltonstall *et al*. 2007). Differences in plasticity of stomatal morphology could further permit a single genotype to acclimate to a range of conditions, making it a strong competitor in heterogeneous environments such as tidal wetlands.

Introduced *Phragmites* produces biomass more quickly, metabolizes carbon and nitrogen more quickly, and it is suspected that the introduced lineage has a photosynthetic advantage over its native conspecific (Mozdzer *et al*. 2013). Using previously identified clonal genotypes (Douhovnikoff and Hazelton 2014), we took advantage of the *g*_max_ framework to investigate variation in stomatal conductance and its dependence on stomatal morphometrics within and between *P. australis* lineages, stands and genets. We quantified maximum stomatal conductance to water, *g*_wmax_, and its plasticity, through measurements of the lengths and densities of stomata on the abaxial (lower) and adaxial (upper) surfaces of leaves. We tested the hypotheses that (i) there are genetic effects on *g*_wmax_ differentiating native and introduced *P. australis* lineages and genotypes and (ii) variation in *g*_wmax_ in response to local site conditions is greater in clones of introduced *P. australis*, indicating greater physiological plasticity that may contribute to the invasive character of this lineage.

## Methods

### Site description

Three marshes in Southern Maine were systematically surveyed for stand scale *P. australis* clonal structure, which was mapped on a 5 × 5 m grid (Douhovnikoff and Hazelton 2014). Marsh sites were Libby (70.310W, 43.563N), Spurwink (70.250W, 43.589N) and the more distant Webhannet (70.585W, 43.286N). Maximum and minimum marsh-to-marsh distances were 43.2 and 5.6 km, respectively. The marshes are back barrier dune systems, and are well suited for comparisons of lineages among stands within the respective marshes; both native and introduced *P. australis* were present, in proximity to each other, at all sites. In the case of the Libby marsh, the introduced and native stands abut each other and overlap in some areas (E. L. G. Hazelton, pers. obs.). The most developed of these sites is the Webhannet marsh, the Spurwink marsh abuts agricultural land and the Libby marsh occupies a watershed with relatively little development or agriculture.

### Sample collection and DNA extraction

Samples were collected in the summer of 2011. The most apical fully expanded leaves were collected from the nearest stem to each sample grid point. Earlier work had determined that the 5 × 5 m sampling grid was ideal for the efficient mapping of genotypic diversity at the sites (Douhovnikoff and Hazelton 2014). Lineages were differentiated by morphological characteristics (Swearingen and Saltonstall 2010), and microsatellite markers (Saltonstall 2003) were used to establish clonal identities (detailed methods in Douhovnikoff and Hazelton 2014).

### Stomatal morphometrics and *g*_wmax_

Leaf material was stored at −20 °C prior to analysis. Epidermal impressions were made using clear nail polish (ethyl acetate) applied directly to the leaf surface, and were mounted on slides. Preliminary measurements indicated that stomatal traits varied systematically along the length of leaves, so middle-adaxial and middle-abaxial leaf surfaces were sampled for consistency. Slides were viewed on Olympus BX-51 microscopes and stomatal morphometrics were determined from images captured at ×400 total magnification using QCapture software (QImaging). ImageJ software (Abramoff *et al*. 2004) was used to count the total number of stomata and measure the lengths of five randomly chosen stomata within a standardized 200 × 200 µm area within each image.

Maximum stomatal conductance to water vapour (mol m^−2^ s^−1^) was calculated using the formula of Brown and Escombe (1900, see also Weyers and Meidner 1990; Franks and Farquhar 2006) parameterized for grass stomata (Taylor *et al*. 2012). Briefly, *g*_wmax_ for each leaf is the sum of maximum conductance values for leaf surfaces (*g*_wmax,*i*_, where *i* is abaxial or adaxial), calculated as:

$${g_{{\rm w}\!\max ,i}} = \displaystyle{d \over v} \times D \times \displaystyle{{{a_{\max }}} \over {l + (\pi {\rm /}2)\sqrt {{a_{\max }}{\rm /}\pi } }}$$

The diffusivity of water in air (*d*, m^2^ s^−1^, at 25 °C), the molar volume of air (*v*, m^3^ mol^−1^, at 25 °C) and *π* are physical and geometric constants. Stomatal density (*D*, m^−2^) and stomatal length (*L*, m) were determined from our measurements and used to derive (i) stomatal size (*S*, m^2^), as 0.25*L*^2^ (stomatal width = 0.25*L*, Taylor *et al*. (2012)); (ii) depth of stomatal pores (*l*, m), as 0.125*L* (equal to guard cell width, Franks *et al*. 2009) and (iii) the maximum stomatal pore area (*a*_max_, m^2^), as 0.4*S* (an empirical relationship for grass stomata determined by Taylor *et al*. 2012). Calculations were made using R Language and Environment (version 3.1.3, R Development Core Team 2015).

### Statistical analysis

We log_e_ transformed *g*_wmax_ prior to statistical analysis. We employed standard approaches for an unbalanced nested 2 × 2 analysis of variance, using the R Language and Environment (version 3.1.3, R Development Core Team 2015), as follows. We performed a Type III conditioning procedure (Fox 2008), initially testing for interactions between the two putative fixed effects, site and lineage, holding the clones as random effects. We detected no significant interactions in the complete data set, though we did find weak but statistically significant interactions when several highly variable clones were excluded from the data. We inferred the significant effects using the complete data set, employing a Type II procedure to ensure full power to determine effects (Langsrud 2003): all factors (site, lineage and clone) exhibited effects with *P*-values <10^−16^. We also employed a more advanced model selection machinery available to Bayesian approaches to calculate the Bayes factors across a wide variety of possible analytic frameworks (Rouder *et al*. 2012), garnering additional support for our choice of analysis. For clones with *N* > 11 ramets, robust estimates of within-clone spatial variation, mean and standard deviation (SD) in log_e_(*g*_wmax_) were made using a permutation test that preserved the variation intrinsic to the data accounting for the variable number of ramets within each clone. This test proceeds by generating two distributions of statistics, a null distribution reflecting the correlation expected under no spatial effect but accounting for unevenness in the underlying spatial distribution of ramets and a corresponding distribution reflecting the correlation observed within the data. The first was generated by randomly permuting which *g*_wmax_ values associate with which (*x*,*y*) position pair for a given ramet, and repeating 10 000 times; for each permutation, a subset of size 10 was taken and a simple Spearman (rank order) correlation was calculated between the pairwise distance between ramets and the difference in their *g*_wmax_ values. The latter distribution was generated to represent the observed data by sampling 10 000 size 10 subsets and again calculating the Spearman correlation. A *P*-value was calculated by finding the fraction of replicates in the observed distribution that were more extreme than all values in the null distribution. While similar in concept to a Mantel test, this permutation approach is significantly more conservative in its *P*-value calculation while still sensitive to even mild (correlation values of 0.1) levels of spatial structure. To ensure that the results were independent of coordinate frame, the test was repeated having rotated the axes by 45°.

## Results

### Site, lineage and clone as factors influencing *g*_wmax_

**Figure 1.**
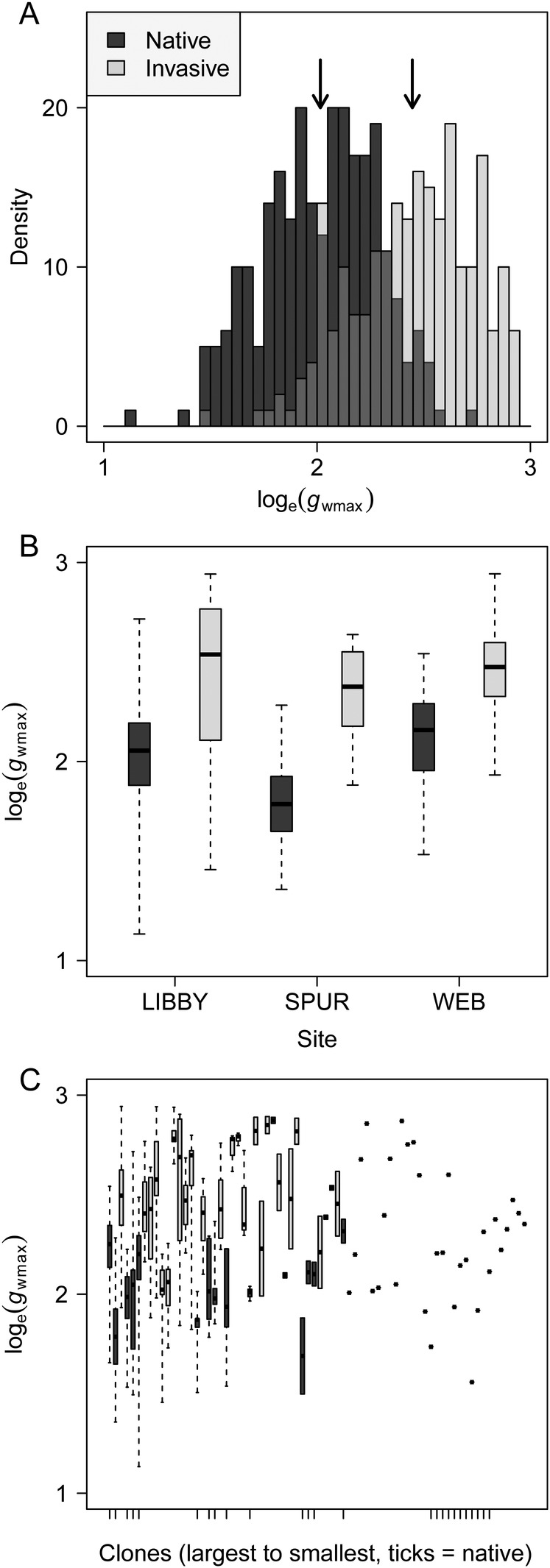
(A) Native and invasive lineages of *P. australis* show significantly different *g*_wmax_ determined on the basis of stomatal morphometrics. (B) Differences in *g*_wmax_ between native and invasive lineages of *P. australis* are consistent between marsh sites in Maine, and are substantially greater than differences in *g*_wmax_ between sites. (C) When comparing unique clones of *P. australis* across three marsh sites in Maine, *g*_wmax_ differentiates clones belonging to native and invasive lineages.

Our model of log_e_(*g*_wmax_) identified significant additive effects of site, lineage and clone (clones having been identified as unique to each site, i.e. completely nested; *F* values 48.06, 495.70 and 4.50 with df = 2, 1 and 68, respectively, *P* < 10^−16^ for all). At the three sites, *P. australis* showed greater mean log_e_(*g*_wmax_) at Webhannet (2.28) and Libby (2.26) than at Spurwink (1.93). When grand means for the native and introduced lineages were compared, log_e_(*g*_wmax_) of the introduced lineage was 21 % greater than the native lineage (Fig. 1A), equivalent to an increase of 54 % when back-transformed to the original scale (mean (2.5–97.5 % quantile): native, 7.5 (4.5–12.1) mol m^−2^ s^−1^; introduced, 11.5 (6.7–18.1) mol m^−2^ s^−1^). This substantial difference between the lineages was relatively consistent across the three sites (16–31 % increase on log_e_ scale depending on site; Fig. 1B). When clones were treated as independent of their classification by site and lineage, and when lineage was excluded from consideration, among-clone variation explained the majority of variance in log_e_(*g*_wmax_) (55 %). Differences among clones were, however, strongly structured by contrasts between native and introduced lineages and sites (Fig. 1C).

### Plasticity (within-clone variation) in *g*_wmax_

**Figure 2.**
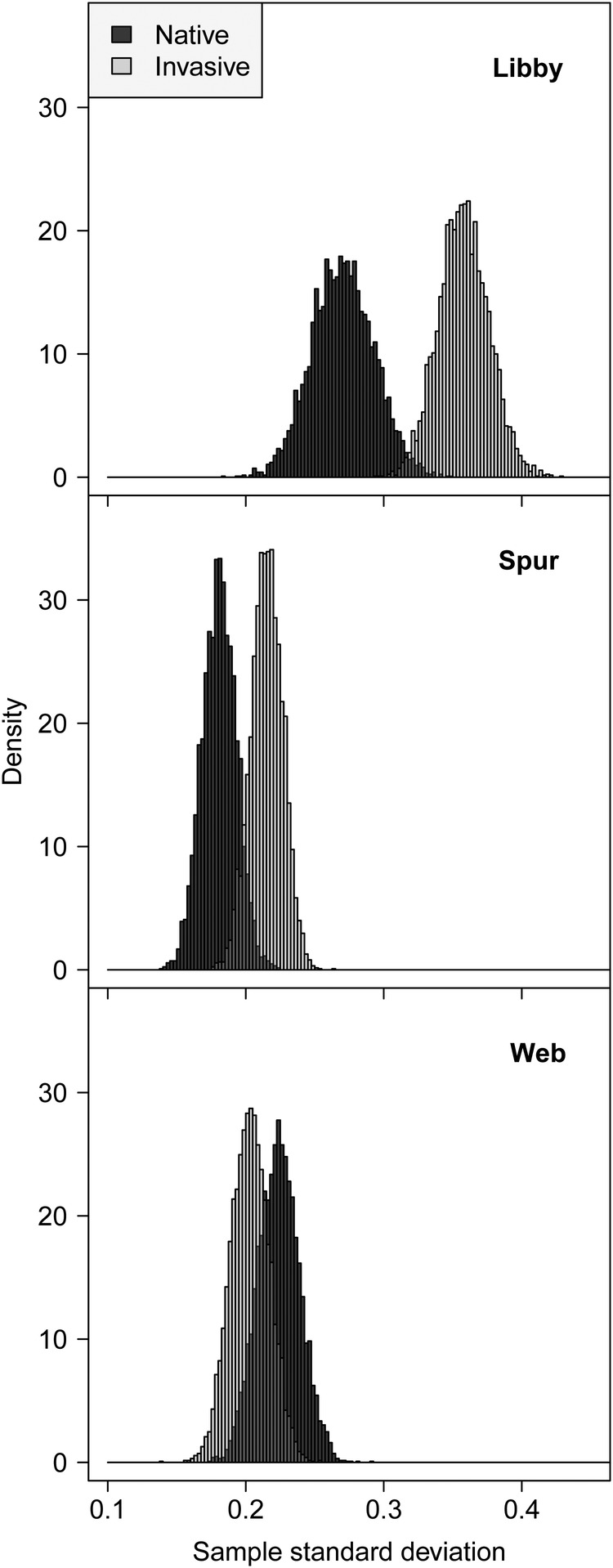
Plasticity (SD) in *g*_wmax_ within the invasive lineage of *P. australis* is similar to, or exceeds, plasticity measured within the native lineage at Libby, Spurwink and Webhannet marshes, in Maine.

Using our entire data set, plasticity in log_e_(*g*_wmax_), determined as the SD of log_e_(*g*_wmax_) conditioned for clone identity (Fig. 2), was greater within the introduced lineage at the Libby (SD: introduced, 0.36; native, 0.27) and Spurwink (SD: introduced, 0.22; native, 0.18) marshes. At the Webhannet marsh, the opposite was true (Fig. 2), but the lineages were also more similar (SD: introduced, 0.20; native, 0.22).

**Figure 3.**
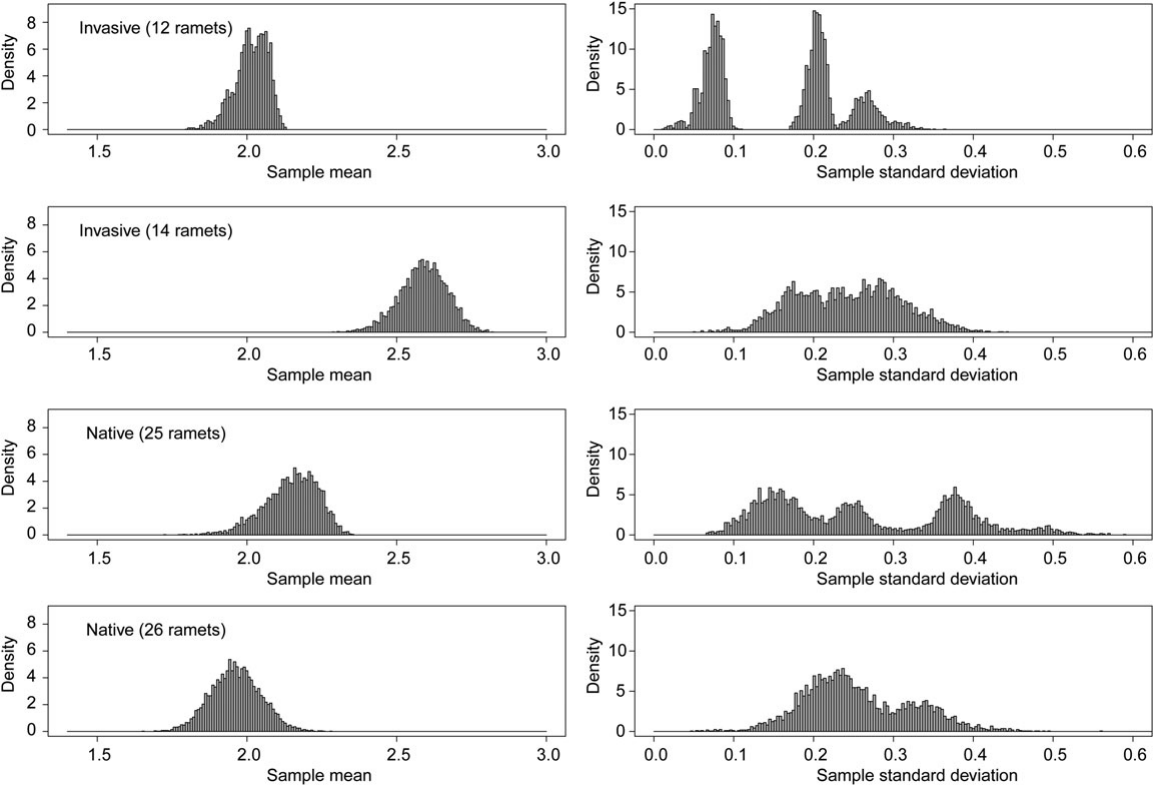
Permutation analysis demonstrates that unimodal distributions for means of *g*_wmax_ within clones of *P. australis* (left column) are linked with multimodal distributions for SDs (right column); clusters of ramets within each genet show unique levels of variability. Results shown are for invasive (BI24, BI51) and native (BN11, BN8) clones at the Libby marsh.

Our investigation of both spatial variation and phenotypic variation in log_e_(*g*_wmax_) within the 10 clones having *N* > 11 ramets found no evidence for significant within-clone spatial structure (permutation test null distribution construction described in Methods with 9999 degrees of freedom, *P* > 0.291). The test used does not rule out spatial autocorrelation as a determinant of finer-scale patterns. Distributions of SDs for log_e_(*g*_wmax_) within large clones at the Libby site, in particular, were multimodal (Fig. 3). The permutation distributions shown in Fig. 3 were realized for each clone by holding the number of ramets to 10 and resampling from the full collection of observed values with replacement: for each clone, 1000 resamplings were made, with the sample mean and sample SD calculated for each sample. This analysis indicates that within these large, genetically homogeneous clones, subsets of ramets showed uniquely identifiable levels of plasticity, perhaps linked by epi-genotype.

### Lineage differences in stomatal morphometrics underpinning *g*_wmax_

**Figure 4.**
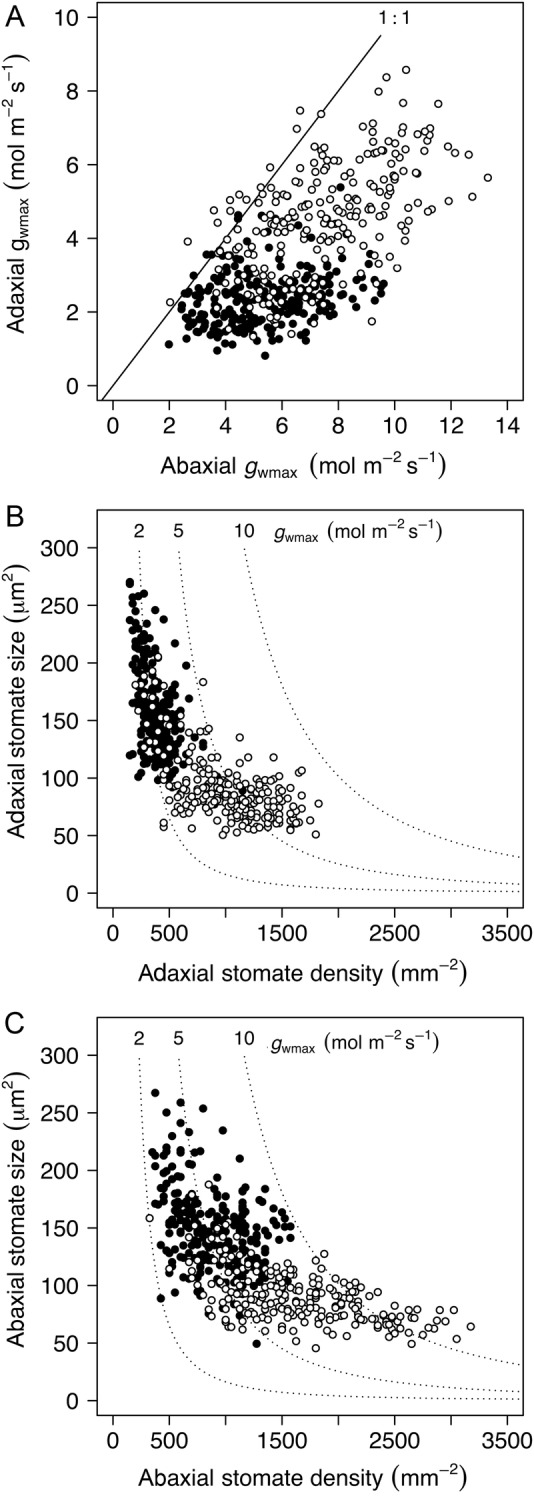
Components of *g*_wmax_ for lineages of *P. australis* native (filled symbols), and invasive (open symbols), to North America. (A) Leaf *g*_wmax_ is the sum of *g*_wmax_ for the adaxial and abaxial leaf surfaces; higher leaf *g*_wmax_ among invasive lineages is a result of increases in both adaxial and abaxial *g*_wmax_. Stomate size shows a negative relationship with stomate density on both the adaxial (B) and abaxial (C) leaf surfaces: higher *g*_wmax_ on abaxial surfaces are linked with greater stomate densities, and the higher stomate densities among invasive *P. australis* are linked with reduced stomate size compared with the native lineage.

The consistently greater *g*_wmax_ of introduced lineages of *Phragmites* was a result of increases in both adaxial and abaxial *g*_wmax_ (Fig. 4A). Size (*S*)–density (*D*) plots indicated that differences in *S* and *D* between the lineages were broadly consistent with a size–density trade-off: the introduced lineage had relatively smaller and more abundant stomata than the native lineage (Fig. 4B and C). Shifts in *S* and *D* among native ramets resulted in conservation of *g*_wmax_ (data for native ramets fall along *g*_wmax_ isoclines in Fig. 4B and C). Among ramets of the introduced lineage, variation in *g*_wmax_ arose from variation in *D* that was not matched by shifts in *S* (Fig. 4B and C).

## Discussion

Previous demonstrations that *g*_wmax_ is reliably linked with gas exchange performance (Dow *et al*. 2014) and demonstrates both heritable variation and environmental plasticity (Franks *et al*. 2009; Fanourakis *et al*. 2015) suggested that simple measurements of the size and spacing of stomata on leaves would provide a strong framework within which to explore phenotypic plasticity in *P. australis*. Our results confirm this expectation; we were able to characterize plasticity in stomatal morphometrics that contributed to differences in *g*_wmax_ between native and invasive lineages. We found that at three marsh sites separated by as much as 43 km, introduced lineages have consistently greater *g*_wmax_ than their native congeners. Thus, *g*_wmax_ can be added to an already extensive list of functional traits that distinguish these genetic variants (stem densities, heights, above ground biomass, leaf area, leaf nitrogen and chlorophyll content, rates of photosynthesis, relative growth rates (RGR) and carbon fixation; reviewed in Mozdzer *et al*. 2013). Our analysis also indicates that plasticity of the introduced lineage, determined as within-genotype variation in *g*_wmax_, is similar to or exceeds that shown by the native lineage. These results provide insights that scale up from stomatal morphometrics to community dynamics.

### Phenotypic variation in stomatal morphometrics

We observed inverse relationships between stomatal size and density, as have been commonly reported in the literature for multiple taxa (Kawamitsu *et al*. 1996; Hetherington and Woodward 2003; Franks *et al*. 2009). The derivation of *g*_wmax_ based on the work of Brown and Escombe (1900) suggests that a trade-off between stomate size and density will be broadly linked with conservation of *g*_wmax_; decreases in stomatal size without a compensatory increase in density should result in decreases in *g*_wmax_ (the relative effect of decreased stomatal size on *g*_wmax_ is smaller when stomata are large because while pore resistance is increased by declines in pore area, parallel decreases in pore depth act to decrease pore resistance; see discussion by Franks *et al*. 2009). We interpret our results as pointing to size–density trade-offs linked with conservation of *g*_wmax_ among leaves from native *P. australis*. Meanwhile, plasticity in *g*_wmax_ among ramets of introduced *P. australis* was linked with greater plasticity in densities of stomata and was sometimes greater than for native clones.

Smaller stomata, as observed for the introduced lineage of *P. australis*, may improve water use efficiency. They are expected to be capable of opening and closing more rapidly (Aasamaa *et al*. 2001; Drake *et al*. 2013); in combination with lower resistance offered by shorter diffusion paths through smaller pores, rapid adjustment should lead to tighter linkage between stomatal responses and the need to regulate transpiration (Knapp 1993). In the case of *P. australis*, improvements in stomatal feedback could allow introduced lineage access to more exposed ground with less reliable water supply, contributing to their observed capacity to reduce soil moisture levels (by accretion, Rooth *et al*. 2003; by transpiration, Windham 2001; by Venturi Effect ventilation, Armstrong and Armstrong 1991). Detailed physiological work assessing the components of leaf gas exchange and hydraulics will be necessary to fully resolve whether differences in water use efficiency are mechanistically linked with stomatal morphometrics in these *Phragmites* lineages.

The *g*_wmax_ values we determined for *P. australis* in Maine, particularly the introduced lineage, were very high (Table 1). They exceeded measurements made by one of the authors in a previous pot-based greenhouse study (Taylor *et al*. 2012). A broad survey of other grass species (Kawamitsu *et al*. 1996) indicates that stomatal morphometrics of cultivated rice (*Oryza sativa*) were most similar to *P. australis*, but *g*_wmax_ values for *P. australis* were higher. This is despite the expectation that a hydrophytic habit and selection for high productivity in rice would be expected to have maximized *g*_wmax_. The *g*_wmax_ values we determined are underpinned by similar stomatal morphometrics to those demonstrated in a previous study that addressed the potential for ploidy level variation of stomatal traits in field collected samples across north-eastern North America (Saltonstall *et al*. 2007; Table 1). Indeed, the stomatal traits reported by Saltonstall *et al*. (2007) suggest even more extreme values for *g*_wmax_ than in our sample (Table 1). Although our study is limited to three marshes in Maine, our results parallel those from a broader set of populations support differences in mean *g*_wmax_ between native and introduced lineages as a general feature of *P. australis*, at least across its north-eastern North American range. Comparison of our measurements, those made by Saltonstall *et al*. (2007), and material of a European origin (Table 1, Taylor *et al*. 2012) also suggests a strong conservation of between-lineage differences in stomatal size while density is more variable (Table 1): plastic responses of *g*_wmax_ in *P. australis* may depend strongly on variation in density of stomata.

**Table 1.** *Phragmites australis* as a species show exceptionally high *g*_wmax_. [1] Saltonstall *et al*. (2007), [2] Taylor *et al*. (2012) and [3] Kawamitsu *et al*. (1996). ^1^Saltonstall *et al*. did not determine lengths of adaxial stomata for most populations, there being no significant difference between surfaces in a subset. ^2^Most extreme among 41 cultivars.

Species/lineage	Mean length of stomata (µm)		Mean density of stomata (mm^−2^)		*g*_wmax_ (mol m^−2^ s^−1^) predicted from mean values	Location, data source
	Adaxial	Abaxial	Adaxial	Abaxial		
*P. australis* native	25	24	394	924	7.94	Maine, this study
*P. australis* invasive	19	19	1002	1635	12.42	
*P. australis* native	25^1^	25	804	1147	12.09	Northeast USA and Canada [1]
*P. australis* invasive	19^1^	19	1726	2167	18.34	
*P. australis*	19	19	419	510	4.38	Glasshouse, UK [2]
*Oryza sativa* cv. Raikei^2^	18	18	646	844	6.65	Japan [3]

*Phragmites australis* is a water-loving species characteristic of marshes and wetlands. Reliable availability of water can relax selection against increases in transpiration (Dudley 1996), allowing for improved net carbon gain or nutrient acquisition (Donovan *et al*. 2007). In hot environments, increased transpiration can improve photosynthetic efficiency and leaf survival by helping to decrease leaf temperatures (Lu *et al*. 1998). In the cool climate of New England, it seems likely that the principal advantage of high stomatal conductances would be to decrease resistance to CO_2_ diffusion into leaves and improve net carbon gain, consistent with observations that the introduced lineage shows greater productivity, responsiveness to carbon enrichment (Mozdzer and Megonigal 2012) and higher RGR, the latter being a proposed factor driving invasion (Mozdzer *et al*. 2013). More broadly, high rates of productivity and the capacity for local habitat modification, e.g. by drying, are traits common to many invasive plants (Cuddington and Hastings 2004); our demonstration that *g*_wmax_ values for introduced *Phragmites* stands exceed those for native stands fits with reports of local drying effects linked the introduced lineage, mediated by both evapotranspiration and sediment accretion (Rooth *et al*. 2003). Summarizing, advantages under a variety of field conditions could arise from increases in transpiration linked with higher *g*_wmax_ that would provide for increased conductance to CO_2_ and reduction in leaf temperature, or improved water use efficiency linked with decreases in stomatal size.

### Community dynamics

High levels of plasticity in stomatal traits support the description of introduced *P. australis* as a ‘Jack-and-master’ of change (Mozdzer and Megonigal 2012; Mozdzer *et al*. 2013). Plasticity in stomatal morphology would be expected to permit a single genotype to acclimate to a range of conditions and make it a strong competitor in a heterogeneous environment. Marsh systems susceptible to Phragmites invasion are starkly heterogeneous in many factors, for example sharp gradients from waterline to bank in salinity, aeration, nutrient availability and water depth (reviewed in Engloner 2009). Comparing North American lineages, Holdredge *et al*. (2010) described a cline ranging from lower elevation associated with waterlogged soils up to higher elevation characterized by high levels of interspecific competition. A single clonal genotype of *P. australis* might span multiple microhabitat transitions in this setting. Genotypes with a plastic localized response at the scale of the ramet could minimize the risks, costs or genetic resources associated with adaptation through sexual reproduction while best optimizing potential opportunities for resource sharing and economies of scale inherent in integrated clonality.

Indeed, ‘Theory predicts that plasticity in … morphologies of plants can transmit heterogeneity from the environment to the population or community’ (Callaway *et al*. 2003). Thus, we can predict that significant variation should be identifiable from the among-lineage down to the among-ramet scales dependent upon local conditions. The lack of spatial structure to our data suggests that drivers of heterogeneity in stands of *P. australis* operate at a scale smaller than the 5 × 5 m scale measured here.

Plasticity is important for both lineages (Mozdzer and Megonigal 2012) and worth comparison against other non-clonal species. However, the lower levels of native plasticity suggest that there may be a cost involved. Net fitness, which synthesizes survival, growth and fecundity, does not necessarily benefit from plasticity (Palacio-López and Gianoli 2011; Pichancourt and Van Klinken 2012). In some circumstances, plasticity can be disadvantageous, for example, when there are costs of inappropriate specialized phenotypes, when environmental cues are unreliable, when the environment is not variable or when the plastic response lags too far behind environmental change (Vretare *et al*. 2001; Callaway *et al*. 2003). Thus, narrower plasticity in the native lineage may constrain optimal microhabitat range or reflect the more homogeneous sites it occupies.

A frequent assertion in invasive plant literature is that phenotypic plasticity is common in invasive species, making possible a broader ecological niche through the expression of site-specific advantageous phenotypes (Richards *et al*. 2006; Davidson *et al*. 2011). Previous work has shown that invading populations have the potential for rapid adaptive evolution (Dlugosch and Parker 2008), which would select for a ‘general purpose genotype’ (Moroney *et al*. 2013). There is some evidence that introduced *P. australis* may be less plastic in its native range (Rolletschek *et al*. 1999) warranting further study of reaction norms in common gardens (e.g. Křiváčková-Suchá *et al*. 2007; Achenbach *et al*. 2012).

After within-genotype variation (plasticity), genetic variation (diversity) was the most important contributor to heterogeneity in phenotypes in this study, with relatively little variation being explained by among-site comparisons. Limited variation among sites may result from an emphasis on clonal reproduction, with limited sexual reproduction, natural selection and genetic drift. Initial models of *P. australis* establishment focussed on the transport of vegetative propagules and would lead to low genet richness at a given site (Bart *et al*. 2006); however, recent research indicates a greater role for sexual reproduction (McCormick *et al*. 2010) with clonal growth clearly important on a local scale (Kettenring and Mock 2012; Douhovnikoff and Hazelton 2014). Instead local genetic diversity can remain relatively high due to long lifespans and mechanisms such as remnant regional dynamics (Douhovnikoff and Hazelton 2014).

## Conclusions

Plasticity in the introduced lineage of *P. australis* is similar to or exceeds that of native stands, both in our results and other reports (Mozdzer and Megonigal 2012; Mozdzer *et al*. 2013). This suggests that capacity for greater plasticity may be a major driver in the introduced lineage's invasiveness. Nonetheless, native *P. australis* does demonstrate considerable plasticity, which may underpin observations of long-term resistance to invasion, resilience and site consolidation. For example, the native lineage is well adapted to both low nutrient environments and exploitation of increasing nitrogen (sensu Hazelton *et al*. 2010). In contrast, the invader consistently outperforms the native in biomass production, nitrogen assimilation and various aspects of carbon metabolism (Mozdzer *et al*. 2013). These differences in physiological traits and trait plasticity may be indicators of different life-history strategies underpinning the ecological success and evolutionary maintenance of the two *P. australis* lineages in North America.

## Sources of Funding

This work was funded by a Bowdoin College internal grant.

## Contributions by the Authors

V.D. was involved in all stages of research execution, data analysis and manuscript preparation. J.O.B. and S.H.T. were involved in data analysis and manuscript preparation. E.L.G.H. and C.S. were involved in research execution and data analysis.

## Conflict of Interest Statement

None declared.

## References

[PLW006C58] Aasamaa K, Sõber A, Rahi M 2001 Leaf anatomical characteristics associated with shoot hydraulic conductance, stomatal conductance and stomatal sensitivity to changes of leaf water status in temperate deciduous trees. Australian Journal of Plant Physiology 28:765–774.

[PLW006C1] Abramoff MD, Magalhaes PJ, Ram SJ 2004 Image processing with ImageJ. Biophotonics International 11:36–42.

[PLW006C2] Achenbach L, Lambertini C, Brix H 2012 Phenotypic traits of *Phragmites australis* clones are not related to ploidy level and distribution range. AoB PLANTS 2012: pls017; 10.1093/aobpla/pls017. 22848787 PMC3407373

[PLW006C3] Alpert P, Simms EL 2002 The relative advantages of plasticity and fixity in different environments: when is it good for a plant to adjust? Evolutionary Ecology 16:285–297. 10.1023/A:1019684612767

[PLW006C4] Armstrong J, Armstrong W 1991 A convective through-flow of gases in *Phragmites australis* (Cav.) Trin. ex Steud. Aquatic Botany 39:75–88. 10.1016/0304-3770(91)90023-X

[PLW006C5] Bastlova D, C˘ížková H, Bastl M, Kvet J 2004 Growth of *Lythrum salicaria* and *Phragmites australis* plants originating from a wide geographical area: response to nutrient and water supply. Global Ecology and Biogeography 13:259–271. 10.1111/j.1466-822X.2004.00089.x

[PLW006C59] Bart D, Burdick D, Chambers R, Hartman JM 2006 Human facilitation of *Phragmites australis* invasions in tidal marshes: A review and synthesis. Wetlands Ecology and Management 14:53–65. 10.1016/j.aquabot.2010.04.003

[PLW006C6] Bellavance M-E, Brisson J 2010 Spatial dynamics and morphological plasticity of common reed (*Phragmites australis*) and cattails (*Typha* sp.) in freshwater marshes and roadside ditches. Aquatic Botany 93:129–134. 10.1016/j.aquabot.2010.04.003

[PLW006C8] Brown HT, Escombe F 1900 Static diffusion of gases and liquids in relation to the assimilation of carbon and translocation in plants. Philosophical Transactions of the Royal Society B: Biological Sciences 193:223–291. 10.1098/rstb.1900.0014

[PLW006C9] Callaway RM, Pennings SC, Richards CL 2003 Phenotypic plasticity and interactions among plants. Ecology 84:1115–1128. 10.1890/0012-9658(2003)084[1115:PPAIAP]2.0.CO;2

[PLW006C10] Clevering OA 1999 The effects of litter on growth and plasticity of *Phragmites australis* clones originating from infertile, fertile or eutrophicated habitats. Aquatic Botany 64:35–50. 10.1016/S0304-3770(99)00009-1

[PLW006C11] Cuddington K, Hastings A 2004 Invasive engineers. Ecological Modelling 178:335–347. 10.1016/j.ecolmodel.2004.03.010

[PLW006C12] Davidson AM, Jennions M, Nicotra AB 2011 Do invasive species show higher phenotypic plasticity than native species and, if so, is it adaptive? A meta-analysis. Ecology Letters 14:419–431. 10.1111/j.1461-0248.2011.01596.x 21314880

[PLW006C13] Dlugosch KM, Parker IM 2008 Founding events in species invasions: genetic variation, adaptive evolution, and the role of multiple introductions. Molecular Ecology 17:431–449. 10.1111/j.1365-294X.2007.03538.x 17908213

[PLW006C14] Donovan LA, Dudley SA, Rosenthal DM, Ludwig F 2007 Phenotypic selection on leaf water use efficiency and related ecophysiological traits for natural populations of desert sunflowers. Oecologia 152:13–25. 10.1007/s00442-006-0627-5 17165094

[PLW006C15] Douhovnikoff V, Dodd RS 2015 Epigenetics: a potential mechanism for clonal plant success. Plant Ecology 216:227–233. 10.1007/s11258-014-0430-z

[PLW006C16] Douhovnikoff V, Hazelton ELG 2014 Clonal growth: invasion or stability? A comparative study of clonal architecture and diversity in native and introduced lineages of *Phragmites australis* (Poaceae). American Journal of Botany 101:1577–1584. 10.3732/ajb.1400177 25253716

[PLW006C17] Dow GJ, Bergmann DC, Berry JA 2014 An integrated model of stomatal development and leaf physiology. New Phytologist 201:1218–1226. 10.1111/nph.12608 24251982

[PLW006C18] Drake PL, Froend RH, Franks PJ 2013 Smaller, faster stomata: scaling of stomatal size, rate of response, and stomatal conductance. Journal of Experimental Botany 64:495–505. 10.1093/jxb/ers347 23264516 PMC3542046

[PLW006C19] Dudley SA 1996 Differing selection on plant physiological traits in response to environmental water availability: a test of adaptive hypotheses. Evolution 50:92–102. 10.2307/2410783 28568873

[PLW006C20] Eller F, Brix H 2012 Different genotypes of *Phragmites australis* show distinct phenotypic plasticity in response to nutrient availability and temperature. Aquatic Botany 103:89–97. 10.1016/j.aquabot.2012.07.001

[PLW006C21] Engloner AI 2009 Structure, growth dynamics and biomass of reed (*Phragmites australis*)—a review. Flora - Morphology, Distribution, Functional Ecology of Plants 204:331–346. 10.1016/j.flora.2008.05.001

[PLW006C22] Fanourakis D, Giday H, Milla R, Pieruschka R, Kjaer KH, Bolger M, Vasilevski A, Nunes-Nesi A, Fiorani F, Ottosen C-O 2015 Pore size regulates operating stomatal conductance, while stomatal densities drive the partitioning of conductance between leaf sides. Annals of Botany 115:555–565. 25538116 10.1093/aob/mcu247 PMC4343285

[PLW006C23] Fox J 2008 Applied regression analysis and generalized linear models, 2nd edn New York, NY: Sage.

[PLW006C24] Franks PJ, Farquhar GD 2006 The mechanical diversity of stomata and its significance in gas-exchange control. Plant Physiology 143:78–87. 10.1104/pp.106.089367 17114276 PMC1761988

[PLW006C25] Franks PJ, Drake PL, Beerling DJ 2009 Plasticity in maximum stomatal conductance constrained by negative correlation between stomatal size and density: an analysis using *Eucalyptus globulus*. Plant, Cell and Environment 32:1737–1748. 10.1111/j.1365-3040.2009.002031.x 19682293

[PLW006C60] Gianoli E, Valladares F 2012 Studying phenotypic plasticity: the advantages of a broad approach. Biological Journal of the Linnean Society 105:1–7.

[PLW006C28] Hansen DL, Lambertini C, Jampeetong A, Brix H 2007 Clone-specific differences in *Phragmites australis*: effects of ploidy level and geographic origin. Aquatic Botany 86:269–279. 10.1016/j.aquabot.2006.11.005

[PLW006C29] Hazelton ELG, Knight TJ, Theodose TA 2010 Glutamine synthetase partitioning in native and introduced salt marsh grasses. Marine Ecology Progress Series 414:57–64. 10.3354/meps08704

[PLW006C30] Hetherington AM, Woodward FI 2003 The role of stomata in sensing and driving environmental change. Nature 424:901–908. 10.1038/nature01843 12931178

[PLW006C31] Holdredge C, Bertness MD, Von Wettberg E, Silliman BR 2010 Nutrient enrichment enhances hidden differences in phenotype to drive a cryptic plant invasion. Oikos 119:1776–1784. 10.1111/j.1600-0706.2010.18647.x

[PLW006C61] Kawamitsu Y, Agata W, Hiyane S, Murayama S, Nose A, Shinjyo C 1996 Relation between leaf gas exchange rate and stomata I. Stomatal frequency and guard cell length in C3 and C4 grass species. Japanese Journal of Crop Science 65:626–633.

[PLW006C32] Kettenring KM, Mock KE 2012 Genetic diversity, reproductive mode, and dispersal differ between the cryptic invader, *Phragmites australis*, and its native conspecific. Biological Invasions 14:2489–2504. 10.1007/s10530-012-0246-5

[PLW006C34] Knapp AK 1993 Gas exchange dynamics in C_3_ and C_4_ grasses: consequence of differences in stomatal conductance. Ecology 74:113–123. 10.2307/1939506

[PLW006C35] Křiváčková-Suchá O, Vávřová P, Čížková H, Čurn V, Kubátová B 2007 Phenotypic and genotypic variation of *Phragmites australis*: a comparative study of clones originating from two populations of different age. Aquatic Botany 86:361–368. 10.1016/j.aquabot.2007.01.001

[PLW006C36] Langsrud O 2003 ANOVA for unbalanced data: use Type II instead of Type III sums of squares. Statistics and Computing 13:163–167. 10.1023/A:1023260610025

[PLW006C37] Lu Z, Percy RG, Qualset CO, Zeiger E 1998 Stomatal conductance predicts yields in irrigated Pima cotton and bread wheat grown at high temperatures. Journal of Experimental Botany 49:453–460. 10.1093/jxb/49.Special_Issue.453

[PLW006C38] Mccormick MK, Kettenring KM, Baron HM, Whigham DF 2010 Spread of invasive *Phragmites australis* in estuaries with differing degrees of development: genetic patterns, Allee effects and interpretation. Journal of Ecology 98:1369–1378. 10.1111/j.1365-2745.2010.01712.x

[PLW006C39] Moroney JR, Rundel PW, Sork VL 2013 Phenotypic plasticity and differentiation in fitness-related traits in invasive populations of the Mediterranean forb *Centaurea melitensis* (Asteraceae). American Journal of Botany 100:2040–2051. 10.3732/ajb.1200543 24107581

[PLW006C40] Mozdzer TJ, Megonigal JP 2012 Jack-and-master trait responses to elevated CO_2_ and N: a comparison of native and introduced *Phragmites australis*. PLoS ONE 7:e42794 10.1371/journal.pone.0042794 23118844 PMC3485286

[PLW006C41] Mozdzer TJ, Brisson J, Hazelton EL 2013 Physiological ecology and functional traits of North American native and Eurasian introduced *Phragmites australis* lineages. AoB PLANTS 5: plt048; 10.1093/aobpla/plt048.

[PLW006C42] Palacio-López K, Gianoli E 2011 Invasive plants do not display greater phenotypic plasticity than their native or non-invasive counterparts: a meta-analysis. Oikos 120:1393–1401. 10.1111/j.1600-0706.2010.19114.x

[PLW006C43] Pichancourt J-B, Van Klinken RD 2012 Phenotypic plasticity influences the size, shape and dynamics of the geographic distribution of an invasive plant. PLoS ONE 7:e32323 10.1371/journal.pone.0032323 22384216 PMC3288080

[PLW006C44] R Development Core Team. 2015 R: a language and environment for statistical computing. Vienna, Austria: R Foundation for Statistical Computing http://www.R-project.org/ (March 2015).

[PLW006C45] Richards CL, Bossdorf O, Muth NZ, Gurevitch J, Pigliucci M 2006 Jack of all trades, master of some? On the role of phenotypic plasticity in plant invasions. Ecology Letters 9:981–993. 10.1111/j.1461-0248.2006.00950.x 16913942

[PLW006C46] Rolletschek H, Rolletschek A, Kühl H, Kohl JG 1999 Clone specific differences in a *Phragmites australis* stand: II. Seasonal development of morphological and physiological characteristics at the natural site and after transplantation. Aquatic Botany 64:247–260. 10.1016/S0304-3770(99)00054-6

[PLW006C48] Rooth JE, Stevenson JC, Cornwell JC 2003 Increased sediment accretion rates following invasion by *Phragmites australis*: the role of litter. Estuaries 26:475–483. 10.1007/BF02823724

[PLW006C49] Rouder JN, Morey RD, Speckman PL, Province JM 2012 Default Bayes factors for ANOVA designs. Journal of Mathematical Psychology 56:356–374. 10.1016/j.jmp.2012.08.001

[PLW006C62] Saltonstall K 2003 Microsatellite variation within and among North American lineages of Phragmites australis. Molecular Ecology 12:1689–1702. 12803624 10.1046/j.1365-294x.2003.01849.x

[PLW006C50] Saltonstall K, Peterson PM, Soreng RJ 2004 Recognition of *Phragmites australis* subsp. americanus (Poaceae: Arundinoideae) in North America: evidence from morphological and genetic analysis. SIDA, Contributions to Botany 21:683–692.

[PLW006C51] Saltonstall K, Glennon K, Burnett A, Hunter RB, Hunter KL 2007 Comparison of morphological variation indicative of ploidy level in *Phragmites Australis* (Poaceae) from Eastern North America. Rhodora 109:415–429. 10.3119/0035-4902(2007)109[415:COMVIO]2.0.CO;2

[PLW006C52] Schulze E, Kelliher FM, Körner C, Lloyd J, Leuning R 1994 Relationships among maximum stomatal conductance, ecosystem surface conductance, carbon assimilation rate, and plant nitrogen nutrition: a global ecology scaling exercise. Annual Review of Ecology and Systematics 25:629–662. 10.1146/annurev.es.25.110194.003213

[PLW006C63] Swearingen J, Saltonstall K 2010 Phragmites field guide: distinguishing native and exotic forms of common reed (*Phragmites australis*) in the United States. Plant Conservation Alliance, Weeds Gone Wild. http://www.nps.gov/plants/alien/fact/pdf/phau1-powerpoint.pdf.

[PLW006C53] Taylor SH, Franks PJ, Hulme SP, Spriggs E, Christin P-A, Edwards EJ, Woodward FI, Osborne CP 2012 Photosynthetic pathway and ecological adaptation explain stomatal trait diversity amongst grasses. New Phytologist 193:387–396. 10.1111/j.1469-8137.2011.03935.x 22040513

[PLW006C54] Thompson K, Hodgson JG, Rich TC 1995 Native and alien invasive plants: more of the same? Ecography 18:390–402. 10.1111/j.1600-0587.1995.tb00142.x

[PLW006C55] Via S, Lande R 1985 Genotype-environment interaction and the evolution of phenotypic plasticity. Evolution 39:505–522. 10.2307/2408649 28561964

[PLW006C56] Vretare V, Weisner SEB, Strand JA, Granéli W 2001 Phenotypic plasticity in *Phragmites australis* as a functional response to water depth. Aquatic Botany 69:127–145. 10.1016/S0304-3770(01)00134-6

[PLW006C64] Weyers JDB, Meidner H 1990 Methods in stomatal research. Harlow: Longman Scientific and Technical.

[PLW006C57] Windham L 2001 Comparison of biomass production and decomposition between *Phragmites australis* (common reed) and *Spartina patens* (salt hay grass) in brackish tidal marshes of New Jersey, USA. Wetlands 21:179–188. 10.1672/0277-5212(2001)021[0179:COBPAD]2.0.CO;2

